# Genistein Suppresses IL-6 and MMP-13 to Attenuate Osteoarthritis in Obese Diabetic Mice

**DOI:** 10.3390/metabo13091014

**Published:** 2023-09-14

**Authors:** Janelle Lopez, Layla Al-Nakkash, Tom L. Broderick, Monica Castro, Brielle Tobin, Jeffrey H. Plochocki

**Affiliations:** 1 Arizona College of Osteopathic Medicine, Midwestern University, Glendale, AZ 85308, USA; 2 Department of Physiology, College of Graduate Studies, Midwestern University, Glendale, AZ 85308, USA; lalnak@midwestern.edu; 3 Laboratory of Diabetes and Exercise Metabolism, Department of Physiology, College of Graduate Studies, Midwestern University, Glendale, AZ 85308, USA; tbrode@midwestern.edu; 4 Department of Anatomy, College of Graduate Studies, Midwestern University, Glendale, AZ 85308, USA; 5 College of Medicine, University of Central Florida, Orlando, FL 32827, USA

**Keywords:** obesity, type 2 diabetes, osteoarthritis, genistein, exercise

## Abstract

Type 2 diabetes mellitus and osteoarthritis (OA) often present as comorbidities. We examined the role of plasma IL-6, chondrocyte MMP-13, and col10a expression in the development of OA in obese diabetic mice. We further investigated dietary genistein and exercise training as potential mitigators of OA. One hundred adult mice (50 females, 50 males) aged 6 weeks were randomized into 5 groups, including lean controls, obese diabetic controls, and obese diabetic mice treated with genistein, exercise training, and genistein plus exercise training. The obese diabetic state was induced by feeding the mice a high-fat, high-sugar diet. Genistein was incorporated into the diet at a concentration of 600 mg genistein/kg. Exercise training was performed on a treadmill and consisted of daily 30 min sessions at 12 m/min, 5 days/week for a 12-week period. After treatment, plasma was collected, and proximal tibias were removed for analysis. Plasma IL-6 and MMP-13 were elevated while col10a was reduced in obese diabetic mice in comparison to lean controls. Dietary genistein treatment reduced IL-6 and MMP-13 expression and increased col10a expression. Histological examination of articular cartilage showed reduced thickness of the uncalcified zones and proteoglycan content in the cartilage of diabetic mice in comparison to mice fed genistein. Exercise training had no significant effect. In conclusion, genistein (and not exercise training) attenuates OA by reducing IL-6 and MMP-13 expression in diabetic mice.

## 1. Introduction

Type 2 diabetes (T2DM) and osteoarthritis (OA) are prevalent diseases that are frequently concomitant. Epidemiologic data show that T2DM is a significant risk factor for OA [1,2,3] while novel experimental data reveal OA is a not only a consequence of mechanical stress but also of metabolic dysregulation [4,5]. Specifically, hyperglycemia associated with T2DM leads to the accumulation of advanced glycation end products and reaction oxygen species that induce the expression of proinflammatory cytokines, proteolytic enzymes, and cartilage matrix destruction characteristic of OA [6,7].

One line of evidence that would further implicate T2DM in the pathogenesis of OA is the altered expression of matrix metalloproteinase-13 (MMP13) and collagen type X (col10a) by chondrocytes in response to interleukin-6 (IL-6) in the diabetic condition. IL-6 is expressed as part of the chronic, low-grade inflammation associated with T2DM [8]. Plasma IL-6 is an independent predictor of T2DM and is significantly elevated in T2DM patients relative to non-diabetic obese controls [9,10]. Chondrocytes in articular cartilage respond to systemic and local IL-6 by upregulating the expression of MMP-13, which leads to articular cartilage degradation associated with OA [11,12]. IL-6 has the opposite effect on col10a by downregulating its expression in chondrocytes, characterizing col10a as an indicator of IL-6 induced changes to cartilage matrix [13,14].

IL-6 and MMP-13 expressions have been suggested as a target in the treatment of OA [15,16]. IL-6 knockout mice have significantly reduced MMP-13 expression and are protected from OA, while IL-6 injections into joint tissue of knockouts causes substantial cartilage degradation, indicating that therapeutics that reduce IL-6 may preserve cartilage tissue [17]. Isoflavones such as genistein may provide one such treatment. Genistein is a naturally occurring isoflavone (found in soy, legumes) that has been shown to have a plethora of biological effects including, but not limited to, beneficial effects on obesity and type 2 diabetes [18]. Relevant to this study, we have previously shown that the same dose of genistein (600 mg genistein/kg) increases bone mass in obese ob/ob hyperglycemic mice and improves facture resistance in mice fed a high-fat and high-sugar diet [19,20]. Dietary intake of isoflavones has also been shown to decrease MMP-13 expression in osteoarthritic cartilages [21]. Exercise is another potential treatment as it also decreases IL-6-induced MMP-13 expression and may help protect cartilage against degeneration [22,23]. However, these treatments have yet to be thoroughly tested in the obese diabetic condition. In this study, we test the hypothesis that IL-6 and MMP-13 are elevated in obese diabetic mice relative to controls and predict that dietary genistein and exercise would reduce plasma IL-6 and cartilage MMP-13 in obese diabetic mice. We further predict that col10a expression would be reduced in diabetic mice and this would be mitigated with genistein and exercise treatment.

## 2. Materials and Methods

Mice of the strain C57BL6 (Charles River Laboratories, Wilmington, MA, USA, n = 50 males, 50 females) aged 6 weeks were randomly assigned to 5 groups. These included (1) lean controls; (2) mice fed a high-fat and high-sugar diet (HFSD) to induce obesity and diabetes; (3) mice fed an HFSD and treated with genistein (Gen); (4) mice fed an HFSD and treated with exercise training (Ex); and (5) mice fed HFSD and treated with dietary genistein and exercise training. The HFSD consisted of chow with 60% fat, 20% carbohydrate, and 20% protein (Dyets Inc., Bethlehem, PA, USA) and drinking water that contained 42 g/L dissolved sugar (55% fructose/45% sucrose). Lean control mice were fed standard chow and normal tap water. Monitoring during this study showed that mice fed the HFSD exhibited hyperglycemia and hyperinsulinemia with a food intake of 2.97 ± 0.52 g/day and sugar uptake of 3.52 ± 0.21 g/d. Genistein was administered at a 600 mg genistein/kg HFSD (Dyets Inc., PA, USA). Exercise training was performed on a treadmill following one week of daily 10 min bouts of acclimation. Training consisted of 30 min sessions at an intensity of 12 m/min, 5 days per day week, for a total of 150 min per week. The treatment period lasted 12 weeks. Mice were housed in an animal facility with a light/dark period of 12 h and temperature maintained at a temperature of 22 °C. Food and water were given ad libitum. The protocol used in this study conformed to the National Institutes of Health’s Guide for the Care and Use of Laboratory Animals and was authorized by the Institutional Animal Care and Use Committee at Midwestern University.

After the 12-week period and 48 h after the last exercise session, mice were sacrificed using 100% CO2 and pneumothorax. Blood was collected via right ventricular puncture. The blood (~0.5–1.0 mL) was immediately transferred to centrifuge tubes (1.5 mL) and spun for 5 min at 5000 rpm. Blood plasma was stored in liquid nitrogen until the IL-6 assay was conducted (Milliplex Assay, Millipore, Billerica, MA, USA), following the manufacturers’ instructions. Proximal tibias were cleaned of soft tissue and fixed in 4% paraformaldehyde. Tibias from one of the limbs was decalcified in 10% EDTA, sectioned in paraffin at a thickness of 10 μm, and stained with H&E. These tibias were then imaged and evaluated for OA. OA was scored according to the criteria in Table 1. Tibias from the other limb, non-decalcified, were processed for immunohistochemical labeling. The tissues for immunohistochemical labeling were sectioned at 10 μm and were incubated in 2.5% normal goat serum prior to incubation of primary antibody to block nonspecific antibody binding. Sections were subsequently incubated with rabbit polyclonal MMP-13 antibody (Proteintech, Rosemont, IL, USA, 18165-1-AP) dilution 1:150 or col10A1 (Biorbyt, Cambridge, UK, orb373500) dilution 1:200 for two days at 4 °C. The secondary antibody, Alexa Fluor 488 Goat anti-Rabbit IgG (H+L) (Life Technologies, Frederick, MD, USA A11008) and DAPI (Invitrogen, Frederick, MD, USA D1306) were applied at 1:800 dilution for one day at 4 °C. Sections were thoroughly rinsed with TBS and placed in refractive index matching solution (RIMS) media for 48 h prior to imaging. RIMS media consisted of 40 gm of Histodenz (Sigma, St. Louis, MO, USA, D2158) in 30 mL of 0.02 M phosphate buffer with 0.1% Tween-20 and 0.01% sodium azide, with pH of 7.5. RIMS was also used as the mounting media. Sections were imaged using ACS APO 40×/1.15 oil (Leica SPE confocal microscope, Leica Microsystems, LAS X 3D, Buffalo Grove, IL, USA) in the blue (405 nm laser line) and green–blue (488 nm laser line) emission spectra to capture DAPI-stained nuclei and MMP-13 or col10a, respectively.

Analysis of the immunofluorescence micrographs was conducted using ImageJ v1.8 (NIH). The color of the images was split into red, blue, and green channels. Using the green channel, which showed only the fluoresced target in grayscale, the tissue was outlined using the selection tool and integrated density was recorded using the “measure” command. Integrated density of both MMP-13 and col10a was measured and calculated as the product of the mean density value of the immunoreactive regions and the area of the tissue. One-way ANOVA was used to test differences among the treatment groups. Significance was set at *p* < 0.05. Data are displayed as mean ± SE.

## 3. Results

**Figure 1:**
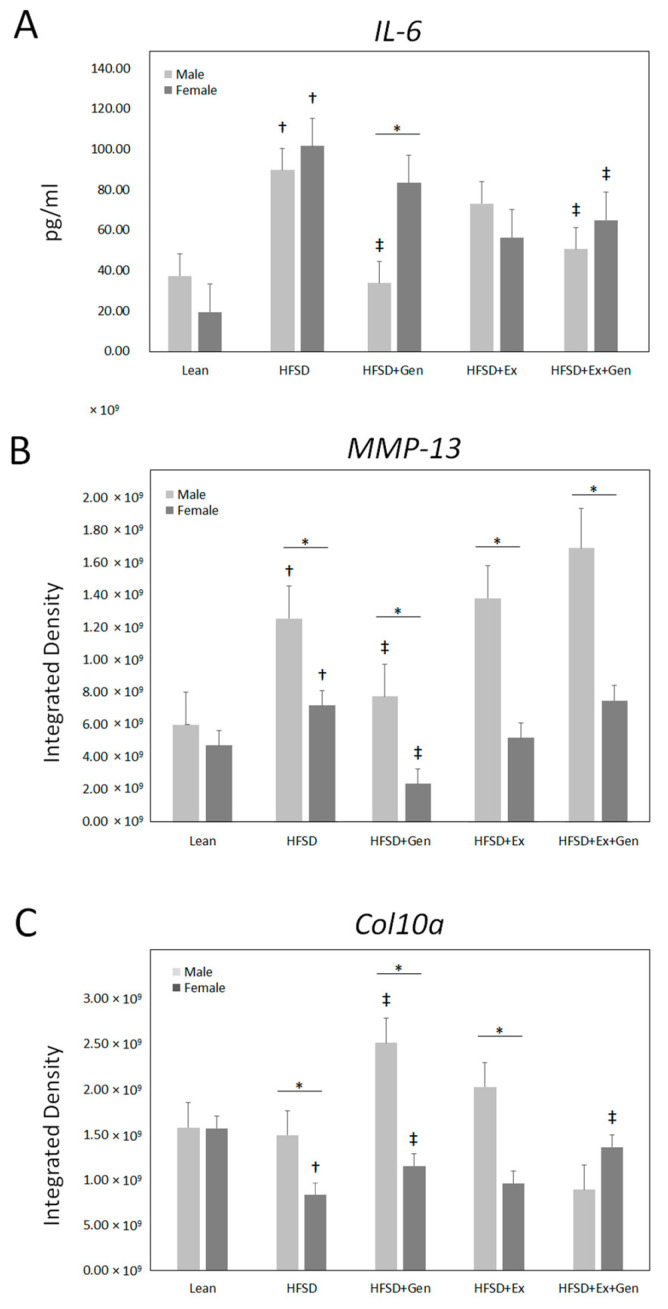
The effects of the HFSD, genistein, and exercise training on (**A**) plasma IL-6, (**B**) chondrocyte MMP-13, and (**C**) chondrocyte col10a expression in male and female mice. Mice were fed a HFSD to induce an obese diabetic state. *, comparison of male and female, *p* < 0.05; †, compared with lean, *p* < 0.05; ‡, compared with an HFSD, *p* < 0.05. HFSD, high-fat and high-sugar diet; Gen, genistein; Ex, exercise training; IL, interleukin; MMP, matrix metalloprotease; col, chondrocyte collagen.

**Figure 2:**
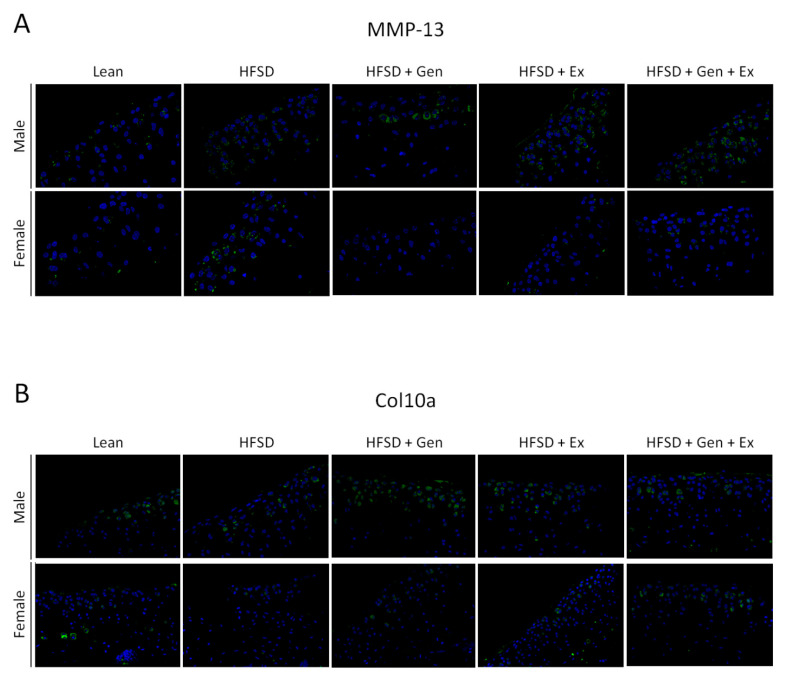
The effects of the HFSD, genistein, and exercise training on immunofluorescence staining for (**A**) MMP-13 and (**B**) col10a in tibial articular cartilage in male and female mice. Mice were fed a HFSD to induce an obese diabetic state. DAPI: blue, MMP-13 and col10a: green. Magnification: 40×. HFSD, high-fat and high-sugar diet; Gen, genistein; Ex, exercise training; MMP, matrix metalloprotease; col, chondrocyte collagen.

The metabolic and phenotypic data from mice in all five groups were reported [24,25]. Mice fed an HFSD displayed obesity, hyperinsulinemia, hyperglycemia, and T2DM [24,25]. Figure 1 illustrates the significant differences in plasma IL-6, chondrocyte MMP-13, and col10a expression in lean mice and mice fed an HFSD. Figure 2 displays immunofluorescence staining of MMP-13 and col10a in articular cartilage of the proximal tibia. There was a significant sex-dependent effect; therefore, data for males and females are presented separately. The HFSD-treated mice had elevated plasma IL-6 in comparison to lean mice of both sexes (Figure 1A). Treatment with dietary genistein reduced IL-6 expression in male, but not female, HFSD-treated mice. Exercise training had no effect on IL-6 in either sex. Treatment with a combination of exercise and genistein reduced plasma IL-6 in HFSD-treated mice of both sexes. Chondrocyte MMP-13 was elevated in HFSD-treated mice in comparison to male and female lean controls. Genistein treatment reduced MMP-13 expression in HFSD-treated mice. Exercise training, alone or in combination with genistein, had no effect in MMP-13 expression in HFSD-treated mice. Expression of MMP-13 was greater in male than female HFSD-treated mice (Figure 1B). Col10a expression was reduced in female but not male HFSD-treated mice in comparison to lean controls. Col10a expression was elevated in male and female HFSD-treated mice treated with dietary genistein in comparison to mice treated with an HFSD alone. It was also elevated in female, but not male, HFSD-treated mice treated with genistein and exercise. Exercise alone had no effect on col10a expression in HFSD-treated mice. Similar to what was observed with MMP-13, HFSD-treated males expressed greater col10a than females (Figure 1C).

**Figure 3:**
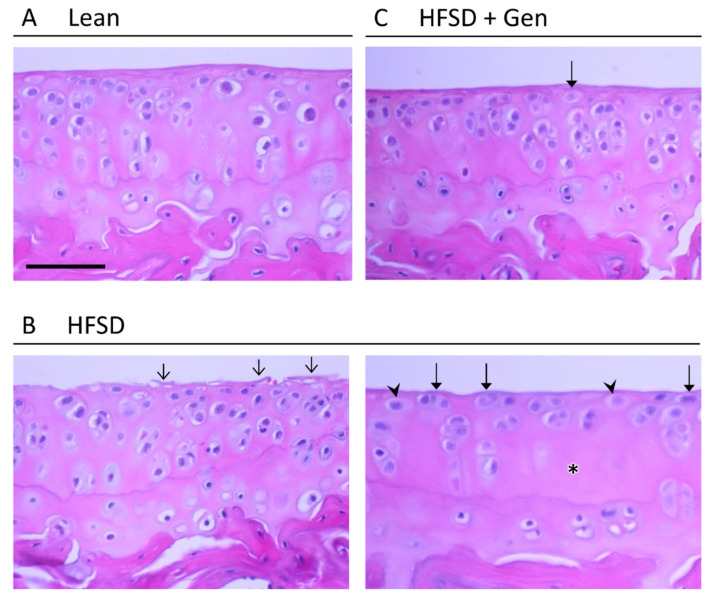
Osteoarthritic changes in knee cartilage are induced with an HFSD and are inhibited by genistein. Articular cartilage of the proximal tibia in (**A**) lean control mice, (**B**) mice fed an HFSD, and (**C**) mice fed an HFSD and treated with dietary genistein. Arrow heads—hypertrophic cells in the tangential zone; thin arrows—fibrillation; thick arrows—cloning in the tangential zone; asterisk—reduced cellularity in the radial zone. Scale bar is 50 μm. H&E, 40×. HFSD, high-fat and high-sugar diet; Gen, genistein.

H&E-stained sections of articular cartilage were assessed for signs of osteoarthritic changes (Figure 3). Cartilage in all male and female lean mice was scored as normal. In comparison, 63% of mice in the HFSD group (seven males, five females) had signs of moderate or severe OA. This included fibrillation, hypertrophic chondrocytes, and reduced matrix staining in the tangential zone; and reduced cellularity in the radial zone. None of the articular cartilage specimens of HFSD-treated mice treated with genistein and/or exercise training exhibited fibrillation, while only three (two males, one female) exhibited mild or moderate OA with hypertrophic cells in the tangential zone and a loss of matrix staining (Figure 3).

## 4. Discussion

The results of our experiment support the hypothesis that diet-induced diabetic obesity results in elevated plasma IL-6, which in turn is associated with elevated MMP-13 and reduced col10a expression by chondrocytes in articular cartilage. Treatment with dietary genistein mitigates the effect of an HFSD by reducing MMP-13 and increasing col10a expression. Exercise training alone is not associated with significant changes in IL-6, MMP-13, or col10a. These findings correspond with histopathological indicators of OA in articular cartilage. Specifically, there is evidence of enhanced degeneration in the joints of both male and female HFSD-fed mice and normal cartilage histology in the joints of HFSD-fed mice also treated with dietary genistein and/or exercise training.

We found significant sex-dependent differences in plasma IL-6, chondrocyte MMP-13, and col10a expression. Cartilage degeneration is mediated by IL-6 in a sex-specific manner [26]. This is largely due to the anti-inflammatory effects of estrogen on joint tissue through the inhibition of proinflammatory cytokines, including IL-6 [27,28]. These effects are observed in adult females prior to menopause and in animals treated with estradiol, which are more resistant to osteoarthritic joint degeneration [29,30]. MMP-13 expression is inhibited by estrogen, which has a prominent role in cartilage matrix degeneration via the IL-6 pathway [31]. This may explain why female mice in our study express less MMP-13 than males and display fewer histological signs of OA. The effects of estrogen on col10a expression with OA have not been extensively investigated, but the changes in col10a expression we observed may also be related to the effect estrogen on col10a [32]. For example, female mice generally produce less col10a than male mice, and this effect is reversed in ovariectomized mice [33].

**Figure 4:**
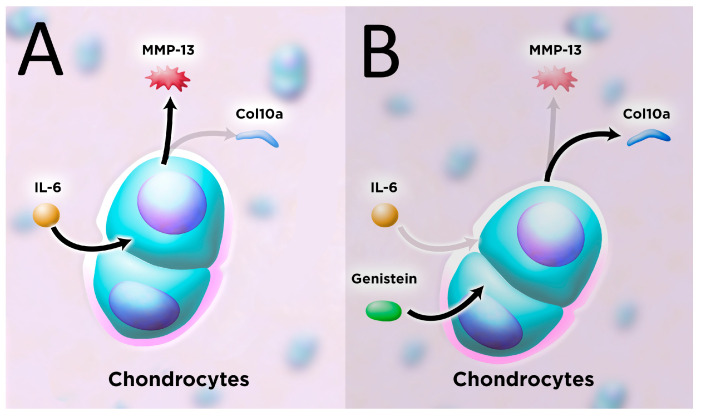
Articular chondrocyte response to IL-6 with (**A**) and without (**B**) genistein treatment (600 mg genistein/kg diet) in obese, hyperglycemic mice. IL-6 increases the expression of MMP-13 and inhibits col10a expression. Treatment with genistein reduces IL-6 and MMP-13 expression and increases col10a expression. Black arrows indicate increased effect and gray arrows indicate inhibited effect. IL, interleukin; MMP, matrix metalloprotease; col, chondrocyte collagen.

Dietary genistein enhanced the degenerative effects of an HFSD, feeding on cartilage health in male and female HFSD-treated mice in our study. Chondrocyte MMP-13 was significantly reduced while col10a was increased in mice treated with genistein (Figure 4). These mice also exhibited healthy articular cartilage with H&E staining. This finding was predicted, as dietary genistein is correlated with improved joint health [34]. The precise mechanism is incompletely understood but may be due to the interruption of IL and TNF-α signaling pathways implicated in the pathogenesis of OA [35]. Those findings, in conjunction with our own, suggest dietary genistein should be explored as a prophylactic treatment in patients with T2DM at risk for OA due to its joint protective properties.

Exercise training in the absence of genistein had no effect on MMP-13 and col10a expression in our study. While the effects of exercise training on col10a expression in cartilage is unknown, treadmill training similar to that in our study has been shown to suppress MMP-13 expression in the joints of healthy rats in a manner that enhances OA [23,36]. However, IL-6 is expressed in muscle tissue during exercise and contributes to increased systemic concentrations [37,38]. It is possible that elevated IL-6 associated with T2DM neutralizes the beneficial effects of exercise on MMP-13 expression. Further research is necessary to elucidate the precise relationship between exercise and OA in the T2DM condition.

There are several limitations of this study. First, we used an animal model that approximates, but may not fully represent, the response to treatment in humans. Caution is warranted when translating these results to human subjects. Second, the effects of genistein and exercise training may be dose dependent. Additional studies are needed to elucidate the precise effects of these treatments on chondrocyte gene expression in males and females. Lastly, there are likely many complicated signaling pathways involved in the expression of col10a and MMP-13 in articular cartilage. This study may have only captured a small portion of the factors involved, so, again, further study is necessary.

## 5. Conclusions

Degeneration of articular cartilage is a clinical concern in patients with T2DM. The results of our investigation support the hypothesis that dietary genistein, a polyphenol with anti-inflammatory properties, attenuates cartilage degeneration in obesogenic diet-induced diabetic mice. We further find that exercise training does not have a significant impact on articular cartilage degeneration in diabetic mice independent of genistein treatment. These data highlight the importance of dietary approaches to promote joint health in diabetic conditions.

## Figures and Tables

**Table 1:** Criteria for scoring OA.

Grade	Osteoarthritic Changes
Normal	Normal structure and staining
Mild	Loss of matrix staining with normal structure
Moderate	Loss of matrix staining, mild fibrillations, hypertrophic chondrocytes
Severe	Loss of matrix staining, deep fibrillations, hypertrophic chondrocytes, reduced cellularity in radial zone

## Data Availability

The datasets used in this study are available from the corresponding author upon request. Data is not publicly available due to privacy.
